# Increased Amygdalar and Hippocampal Volumes in Young Adults with Social Anxiety

**DOI:** 10.1371/journal.pone.0088523

**Published:** 2014-02-11

**Authors:** João Paulo Machado-de-Sousa, Flávia de Lima Osório, Andrea P. Jackowski, Rodrigo A. Bressan, Marcos H. N. Chagas, Nelson Torro-Alves, André L. D. DePaula, José A. S. Crippa, Jaime E. C. Hallak

**Affiliations:** 1 Department of Neuroscience and Behavior, Ribeirão Preto Medical School, University of São Paulo (USP), Ribeirão Preto – SP, Brazil; 2 Department of Psychiatry, Federal University of São Paulo (UNIFESP), São Paulo – SP, Brazil; 3 Department of Psychology, Federal University of Paraíba (UFPB), João Pessoa – PB, Brazil; University of Electronic Science and Technology of China, China

## Abstract

**Background:**

Functional neuroimaging studies have consistently shown abnormal limbic activation patterns in socially anxious individuals, but structural data on the amygdala and hippocampus of these patients are scarce. This study explored the existence of structural differences in the whole brain, amygdala, and hippocampus of subjects with clinical and subthreshold social anxiety compared to healthy controls. We hypothesized that there would be volumetric differences across groups, without predicting their direction (i.e. enlargement or reduction).

**Methods:**

Subjects classified as having social anxiety disorder (n = 12), subthreshold social anxiety (n = 12) and healthy controls (n = 14) underwent structural magnetic resonance imaging scans. The amygdala and hippocampus were defined *a priori* as regions of interest and volumes were calculated by manual tracing. Whole brain volume was calculated using voxel-based morphometry.

**Results:**

The bilateral amygdala and left hippocampus were enlarged in socially anxious individuals relative to controls. The volume of the right hippocampus was enlarged in subthreshold social anxiety participants relative to controls. No differences were found across groups in respect to total brain volume.

**Conclusions:**

Our results show amygdalar and hippocampal volume alterations in social anxiety, possibly associated with symptom severity. The time course of such alterations and the cellular and molecular bases of limbic plasticity in social anxiety should be further investigated.

## Introduction

Neuroimaging studies have identified a number of brain structures underlying the behavioral manifestations of social anxiety, including mainly the prefrontal and anterior cingulate cortices and limbic and paralimbic structures, with an emphasis on the amygdala [1].

There is reasonable consensus today that amygdala hyper-responsiveness is a core feature of social anxiety. The amygdala has been implicated in the acquisition of conditioned fear [2] and, together with the hippocampus, which is believed to process contextual cues, the amygdala is central to the classification of social stimuli as potentially threatening and to the elaboration of early responses to them.

Neuroimaging has provided consistent evidence concerning the function of limbicareas in social anxiety. Based on the vast literature describing functional alterations underlying social anxiety (for a review, please see Freitas-Ferrari et al., 2010 [1]), recent studies using resting-state magnetic resonance imaging (MRI) have described abnormal connectivity between the amygdala and areas associated with the processing of socially relevant information, including the orbitofrontal, prefrontal, and visual cortices [3], [4].

Despite the abundance of functional studies, structural data concerning the same regions are scarce. An early investigation failed to find volumetric differences in the caudate, putamen, thalamus, and whole brain between patients with social anxiety disorder (SAD) and healthy controls [5]. Recently, however, Irle et al. (2010) [6] found decreased amygdalar and hippocampal volumes in men with generalized SAD relative to controls and Liao et al. (2011) [7] reported reduced gray matter volumes in the right hippocampus and inferior temporal gyrus in SAD, which were associated with enhanced resting-state functional connectivity. Finally, in the last and most recent article describing structural changes associated with social anxiety, Syal et al. (2012) [8] described cortical thickness reductions in areas surrounding the fusiform and post-central gyri and, specifically on the right hemisphere, in the frontal, temporal, parietal, and insular cortices. Their volumetric analyses, however, showed no differences between patients with SAD and healthy controls in respect to the volume of the amygdala and hippocampus.

The findings of increased activity and decreased volume in the amygdala and hippocampus in SAD are intriguing. Although the relationship between activity and volume of brain structures is not yet clear, some authors suggest that increased metabolic activity is likely to be associated with increased blood flow, which, in turn, might result in subtle volume increases [9]. Along the same line, Supekar et al. (2010) [10] proposed that increased gray matter volume could reflect enhanced synaptic connectivity. If this is true, in agreement with the repeated observations of enhanced amygdala activity in SAD, we could expect amygdala volumes to be enlarged in socially anxious individuals, and not reduced as the only two structural studies available have reported.

Another point to be considered in investigating volume changes in limbic structures is the role of stress. Because of the nature of their fears, socially anxious individuals are subject to increased stress in daily life compared to non-anxious people, and there is evidence that neurons in the amygdala and hippocampus may suffer excitotoxic damage resulting from sustained glucocorticoid activity associated with stress [11], [12]. Accordingly, volume reductions in limbic structures would be an expected finding in SAD, at least in patients with a longer disease duration.

With these considerations in mind, we decided to compare the volumes of the amygdala, hippocampus, and whole brain of treatment-naïve individuals meeting criteria for social anxiety disorder and subthreshold social anxiety and healthy controls. We hypothesized that amygdalar and hippocampal volumes would be different across the three groups, without predicting, however, the direction of potential differences.

Our data showed that the bilateral amygdala and hippocampus are enlarged in socially anxious subjects compared to controls. Specificities of these findings are described and discussed below.

## Methods

### Sample

Subjects were randomly selected among the participants of a previous investigation on the prevalence of SAD involving 2.319 university students assessed with SAD screening instruments [13]. A subgroup of 60 volunteers from the original sample were invited to attend an individual interview with an experienced psychiatrist for diagnostic (or healthy status) confirmation with the Brazilian version [14], [15] of the Structured Clinical Interview for the Diagnostic and Statistical Manual of Mental Disorders, 4^th^ edition (DSM-IV)– clinical and non-patient versions(SCID-CV and SCID-NP)[16].

According to the results of the assessment, 40 participants were invited to take part in the study and assigned to three groups: social anxiety disorder (SAD, n = 13), subthreshold social anxiety (SSA, n = 13), and healthy non-anxious subjects (NSA, n = 14). Participants were classified as having social anxiety disorder when they fulfilled DSM-IV criteria, and subthreshold social anxiety when unreasonable fear of a social situation was present but without associated avoidance or impairment, as proposed by Crum and Pratt (2001)[17]. Data from three subjects (two from the SAD and one from the SSA group) were not included in the analysis because their MRI scans were deemed inadequate by the radiology staff; therefore, the final SAD group had 11 participants and the SSA group had 12. The three groups were matched according to age, sex, education, and socioeconomic status and all subjects were right-handed.

We did not include participants with organic brain syndromes or relevant general medical conditions identified during the interview and clinical examination, epilepsy, psychiatric disorders other than SAD (SAD and SSA groups), history of drug abuse (except nicotine) or who were pregnant at the time of assessment.

Participants in the three groups were not using psychotropic medications and had never received pharmacological or psychotherapeutic treatment for any psychiatric disorder.

### Ethics statement

The study protocol was approved by the Ethics Committee of the Ribeirão Preto Medical School University Hospital (Process HCRP #11194) and all volunteers gave their signed informed consent to participate.

### MRI data acquisition

MRI scans were conducted by the radiology staff of the Ribeirão Preto Medical School University Hospital using a 1.5 T Siemens Magneton Vision (Erlangen, Germany) unit with a 25 mT head gradient coil and a circularly polarized coil. To minimize the effects of head movements, subjects were positioned at the scanner by the same staff member using the orbitomeatal line as reference.

A T1-gradient echo volumetric sequence was acquired in the sagittal plane for the multiplanar reformatting and for ROI and voxel-based morphometry (VBM) analyses (TR = 9.7 ms, TE = 4 ms, flip angle  = 12°, FOV = 256 mm with continuous 1-milimeter slices in a total of 160 slices per block, matrix  = 256×256, NEX = 1). All images were examined by a neuroradiologist and considered adequate.

### Image processing

The images were initially processed with the software ANALYZE AVW 7.0 [18]. The hippocampus and the amygdala were manually traced according to detailed directions proposed by Schummanet al. (2004) [19]. The images of the T1-weighted sequence were converted into 0.5 mm^3^ voxels and reoriented according to the hippocampal axis (horizontal axis parallel to a line crossing the rostral and caudal poles of the hippocampus). Manual tracing was done over oblique coronal slices and complementary checked on the sagittal and axial planes. Random repeated measures were made from 10 subjects and yielded an intraclass inter-rater reliability coefficient >0.96 for the bilateral hippocampus and >0.95 for the bilateral amygdala.

To compensate for possible differences in brain volume across subjects, the volumes of the amygdala and hippocampus were corrected by dividing the volumes of these structures by the volume of the whole brain of each participant.

Total brain volume was measured through VBM analysis using the VBM Toolbox of the software Statistical Parametric Mapping 5 (SPM5 - dbm.neuro.uni-jena.de/vbm). The sum of all voxels within the segmented images was similar to the total volume of the corresponding partition. The total volume of the brain was then calculated through the sum of gray and white matter volumes.

### Statistical analysis

Clinical and demographic data were analyzed using Student's *t* test for continuous variables and chi-square tests for nominal variables. Volumetric data were compared using analysis of variance (ANOVA) followed by Duncan's post hoc tests when there were differences across the three groups.

## Results

We found no statistically significant differences among the SAD, SSA, and NSA groups in terms of their socio-demographic characteristics, as shown in Table 1.

**Table 1:** Demographic characteristics of groups SAD, SSA, and NSA.

	Group					
	SAD	SSA	NSA			
**N**	12	12	14			
				x_2_	df	*p*
**Gender (M/F)**	7/5	8/4	11/3	1.25	2	0.54
**Education (years)**				3.63	2	0.73
**12**	4	2	5			
**13**	5	7	4			
**14**	2	3	4			
**15**	1	0	1			
				F	df	*p*
**Age**	20.17	20.83	19.79	1.91	2	0.16

SAD =  social anxiety disorder; SSA =  subthreshold social anxiety; NSA =  non-socially anxious controls

**Figure 1:**
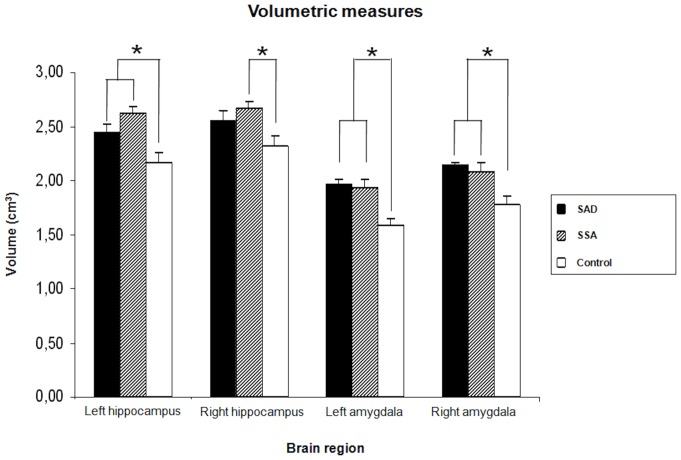
Volumetric differences in the amygdala and hippocampus of participants with social anxiety disorder (SAD), subthreshold social anxiety (SSA), and non-anxious controls. (*) Indicate statistically significant differences between groups.

In respect to the volumetric analysis, the volumes of the whole brain (p = 0.76) and of gray (p = 0.84) and white matter (p = 0.79) were equivalent across groups; there were, however, specific differences in the amygdala and hippocampus. Post hoc tests revealed volume increases in the bilateral amygdala (rAMG: F_2;34_ = 8.66; p = 0.001; lAMG: F_2;34_ = 11.33; p<0.001) and left hippocampus (F_2;34_ = 10.20; p<0.001) of SAD and SSA participants compared to healthy controls. The volume of the right hippocampus was significantly increased in the SSA group compared to controls (F_2;34_ = 4.50; p<0.02). The mean volumes of all brain structures examined in the three groups are presented in Table 2. There were no differences between the SAD group and the other two groups regarding right hippocampal volume. Figure 1 provides a graphic representation of these differences.

**Table 2:** Mean volumes (cm^3^) of right and left amygdala, hippocampus, white and gray matter, and whole brain in subjects with social anxiety (SA), subthreshold social anxiety (SSA) and non-anxious healthy controls (NSA).

Brain structure		Mean volume (cm^3^) ± SD per group				
		SAD (n = 11)	SSA (n = 12)	NSA (n = 14)	F_2,34_	*p*
Amygdala	L	1.96±0.20	1.93±0.30	1.59±0.20	<0.001	11.332
	R	2.14±0.07	2.08±0.27	1.78±0.30	0.001	8.660
Hippocampus	L	2.45±0.20	2.62±0.22	2.16±0.33	<0.001	10.205
	R	2.55±0.30	2.67±0.22	2.31±0.40	0.018	4.509
Gray matter		780.76±79.0	769.41±52.27	783.29±58.46	0.845	0.169
White matter		454.26±57.60	435.14±57.03	452.11±94.30	0.786	0.242
Whole brain		1235.02±113.71	1204.55±94.77	1235.40±136.40	0.760	0.276

L =  left; R =  right.

## Discussion

This study investigated the existence of structural abnormalities in the amygdala and hippocampus of treatment-naïve socially anxious individuals in relation to healthy controls using MRI. As hypothesized, we found volumetric alterations in subjects with clinical and sub-clinical social anxiety compared to volunteers with no social anxiety. Specifically, socially anxious participants had increased bilateral amygdala and left hippocampus volumes. The right hippocampus was also enlarged in the group with sub-clinical social anxiety compared to controls.

Only two previous studies examined the same brain structures in social anxiety. While Syal et al. (2012) [8] found no volumetric differences in the amygdala and hippocampus of subjects with SAD and controls, Irle et al. (2011) [6] reported precisely the opposite to our findings; that is, volume reductions in the amygdala and hippocampus of subjects with generalized SAD relative to controls, which led us to take a closer look at the processes of atrophy/hypertrophy of limbic structures possibly associated with anxiety.

Research on stress-induced brain plasticity has shown that chronic immobilization stress increases anxiety-like behavior in rats and that this is accompanied by dendritic hypertrophy in the basolateral amygdala and dendritic atrophy in hippocampal area CA3 [20]. In the molecular level, dendritic architecture is mediated by BDNF, the expression of which has been shown to be reduced in area CA3 and increased in the basolateral amygdala as a result of chronic immobilization stress, mirroring the structural changes described and following the same temporal profiles of reversal after stress cessation [21].

Considering that social anxiety implies chronic stress, it is possible that the same mechanisms apply to the brain of socially anxious humans, whose amygdala could be enlarged as a result of brain-derived neurotrophic factor (BDNF) over-expression leading to dendritic hypertrophy. Even though this interpretation might be correct, our finding of unilateral hippocampal enlargement in subjects with subthreshold social anxiety relative to controls remains unexplained.

A closer analysis of the samples enrolled here and in the study by Irle et al. (2010) [6] might help reduce the discrepancy between findings and shed light into limbic plasticity processes underlying social anxiety. While the mean age of our participants was around 20 years in the three groups, participants in the study by Irle et al. (2010) [6] were on average 10 years older. This is of particular importance because prolonged stress has been linked to brain atrophy - especially in the hippocampus – believed to result from chronic exposure to glucocorticoids [12], whose receptor density is high in the amygdala and hippocampus [22]. Interestingly, the study by Syal et al. (2012) [8] describing cortical thinning in the brain of socially anxious subjects also involved a sample whose mean age was very similar to – and somewhat higher than – the sample studied by Irle et al. (2010) [6].

Although glucocorticoid excitotoxicity is a strong candidate to explain volume reductions in limbic structures in individuals suffering from social anxiety disorder for long periods, it is not the only one. One typical feature of SAD is the high rate of co-occurring psychiatric conditions, the most common of which is depression [23]. It seems beyond doubt that depression is associated with hippocampal atrophy [24], [25] and a relatively recent meta-analysis has shown that *unmedicated* depression is linked with amygdalar atrophy [26].

Taken together, evidence from neuroimaging, cellular, and molecular studies show that our findings and those of Irleet al. (2010) [6] are not necessarily discrepant and can actually be combined in a more comprehensive hypothesis postulating that limbic - and especially amygdalar - plasticity in SAD is biphasic, with volume increases in early stages followed by atrophy resulting from excitotoxic processes in the long run.

The possibility also exists that the volume of limbic structures may be affected by disorder severity instead of or in complex interaction with age, and it could be the case that progressive volume reductions actually result from increased disorder severity. Unfortunately, we did not include measures of disorder severity in our study and were thus unable to investigate this interaction.

Our study has other limitations that should be taken into consideration in interpreting our findings. Although we recruited only subjects who had not been diagnosed with SAD before and thus had never received any psychological or pharmacological treatment for the disorder; we did not specify the SAD subtype (specific or generalized) of our socially anxious participants or how many of them had indicators of depression. In respect to our methods, ROI-based analyses have been reported to increase the risk of false positive and negative results [1] that cannot be ruled out and future studies should consider the inclusion of whole-brain VBM or other automated techniques to check for alterations in non-specific regions.

## Conclusion

We found structural abnormalities in the amygdala and hippocampus of socially anxious individuals compared to healthy controls. Specifically, subjects with clinical and subthreshold social anxiety had increased bilateral amygdala and left hippocampus volume.

Combined with results from the only other investigation available on the morphology of the amygdala and hippocampus in social anxiety, our findings suggest that limbic plasticity underlying social anxiety has a biphasic pattern characterized by increased amygdalar and hippocampal volume in the early stages of the disorder followed by sustained volume reductions over time.

Further longitudinal structural neuroimaging research is warranted to test this hypothesis and establish the direction of morphological alterations in SAD and their time course and possible interactions with disorder severity. In the molecular level, investigators should look at processes responsible for the atrophy/hypertrophy of limbic structures, with an emphasis on the role of BDNF and the relationship between altered activation and volume.

## References

[1] Freitas-Ferrari MC, Hallak JE, Trzesniak C, Filho AS, Machado-de-Sousa JP, et al (2010) Neuroimaging in social anxiety disorder: a systematic review of the literature. Prog Neuropsychopharmacol Biol Psychiatry 34: 565–580. 20206659 10.1016/j.pnpbp.2010.02.028

[2] Phillips RG, LeDoux JE (1992) Differential contribution of amygdala and hippocampus to cued and contextual fear conditioning. Behav Neurosci 106: 274–285. 1590953 10.1037//0735-7044.106.2.274

[3] Liao W, Qiu C, Gentili C, Walter M, Pan Z, et al (2010) Altered effective connectivity network of the amygdala in social anxiety disorder: a resting-state FMRI study. PLoS One 22: e15238. 10.1371/journal.pone.0015238 PMC3008679 21203551

[4] Hahn A, Stein P, Windischberger C, Spindelegger C, Moser E, et al (2011) Reduced resting-state functional connectivity between amygdala and orbitofrontal cortex in social anxiety disorder. Neuroimage 56: 881–889. 21356318 10.1016/j.neuroimage.2011.02.064

[5] Potts NL, Davidson JR, Krishnan KR, Doraiswamy PM (1994) Magnetic resonance imaging in social phobia. Psychiatry Res 52: 35–42. 8047620 10.1016/0165-1781(94)90118-x

[6] Irle E, Ruhleder M, Lange C, Seidler-Brandler U, Salzer S, et al (2010) Reduced amygdalar and hippocampal size in adults with generalized social phobia. J Psychiatry Neurosci 35: 126–131. 20184810 10.1503/jpn.090041 PMC2834794

[7] Liao W, Xu Q, Mantini D, Ding J, Machado-de-Sousa JP, et al (2011) Altered gray matter morphometry and resting-state functional and structural connectivity in social anxiety disorder. Brain Res 4: 167–177. 10.1016/j.brainres.2011.03.018 21402057

[8] Syal S, Hattingh CJ, Fouché JP, Spottiswoode B, Carey PD, et al (2012) Gray matter abnormalities in social anxiety disorder: a pilot study. Metab Brain Dis 27: 299–309. 22527992 10.1007/s11011-012-9299-5

[9] Frodl T, Meisenzahl EM, Zetzsche T, Born C, Jäger M, et al (2003) Larger amygdala volumes in first depressive episode as compared to recurrent major depression and healthy control subjects. Biol Psychiatry 53: 338–344. 12586453 10.1016/s0006-3223(02)01474-9

[10] Supekar K, Uddin LQ, Prater K, Amin H, Greicius MD, et al (2010) Development of functional and structural connectivity within the default mode network in young children. Neuroimage 52: 290–301. 20385244 10.1016/j.neuroimage.2010.04.009 PMC2976600

[11] Sheline YI, Gado MH, Price JL (1998) Amygdala core nuclei volumes are decreased in recurrent major depression. Neuroreport 13: 2436. 10.1097/00001756-199806220-00021 9674587

[12] Sapolsky RM, Uno H, Rebert CS, Finch CE (1990) Hippocampal damage associated with prolonged glucocorticoid exposure in primates. J Neurosci 10: 2897–2902. 2398367 10.1523/JNEUROSCI.10-09-02897.1990 PMC6570248

[13] Baptista CA, Loureiro SR, de Lima Osório F, Zuardi AW, Magalhães PV, et al (2012) Social phobia in Brazilian university students: prevalence, under-recognition and academic impairment in women. J Affect Disor 136: 857–861. 10.1016/j.jad.2011.09.022 22018945

[14] Del-Ben CM, Vilela JAA, Crippa JAS, Hallak JEC, Labate CM, et al (2001) Test retest reliability of the Structured Clinical Interview for DSM-IV – Clinical Version (SCID-CV) translated into Portuguese. Rev Bras Psiquiatr 23: 156–159.

[15] Crippa JA, de Lima Osório F, Del-Ben CM, Filho AS, da Silva Freitas MC, et al (2008) Comparability between telephone and face-to-face structured clinical interview for DSM-IV in assessing social anxiety disorder. Perspect Psychiatr Care 44: 241–247. 18826462 10.1111/j.1744-6163.2008.00183.x

[16] First MB, Spitzer RL, Gibbon M, Williams JBW (1997) Structured Clinical Interview for DSM-IV Axis I Disorders – Clinical Version (SCID-CV). Washington DC: American Psychiatric Press.

[17] Crum RM, Pratt LA (2001) Risk of heavy drinking and alcohol use disorders in social phobia: a prospective analysis. Am J Psychiat 158: 1693–1700. 11579004 10.1176/appi.ajp.158.10.1693

[18] Robb RA, Hanson DP, Karwoski RA, Larson AG, Workman EL, et al (1989) ANALYZE: A comprehensive, operator-interactive software package for multidimensional medical image display and analysis. Comput Med Imaging Graph 13: 433–454. 2688869 10.1016/0895-6111(89)90285-1

[19] Schumann CM, Hamstra J, Goodlin-Jones BL, Lotspeich LJ, Kwon H, et al (2004) The amygdala is enlarged in children but not adolescents with autism; the hippocampus is enlarged at all ages. J Neurosci 24: 6392–6401. 15254095 10.1523/JNEUROSCI.1297-04.2004 PMC6729537

[20] Vyas A, Pillai AG, Chattarji S (2004) Recovery after chronic stress fails to reverseamygdaloid neuronal hypertrophy and enhanced anxiety-like behavior. Neurosci 128: 667–673. 10.1016/j.neuroscience.2004.07.013 15464275

[21] Lakshminarasimhan H, Chattarji S (2012) Stress leads to contrasting effects on the levels of brain derived neurotrophic factor in the hippocampus and amygdala. PLoS One 7: e30481. 22272355 10.1371/journal.pone.0030481 PMC3260293

[22] Johnson LR, Farb C, Morrison JH, McEwen BS, LeDoux JE (2005) Localization of glucocorticoid receptors at postsynaptic membranes in the lateral amygdala. Neuroscience 136: 289–299. 16181741 10.1016/j.neuroscience.2005.06.050

[23] Chagas MH, Nardi AE, Manfro GG, Hetem LA, Andrada NC, et al (2010) Guidelines of the Brazilian Medical Association for the diagnosis and differential diagnosis of social anxiety disorder. Rev Bras Psiquiatr 32: 444–452. 21308267 10.1590/s1516-44462010005000029

[24] Campbell S, Marriott M, Nahmias C, MacQueen GM (2004) Lower hippocampal volume in patients suffering from depression: a meta-analysis. Am J Psychiatry 161: 598–607. 15056502 10.1176/appi.ajp.161.4.598

[25] Videbech P, Ravnkilde B (2004) Hippocampal volume and depression: a meta-analysis of MRI studies. Am J Psychiatry 161: 1957–1966. 15514393 10.1176/appi.ajp.161.11.1957

[26] Hamilton JP, Siemer M, Gotlib IH (2008) Amygdala volume in major depressive disorder: a meta-analysis of magnetic resonance imaging studies. Mol Psychiatry 13: 993–1000. 18504424 10.1038/mp.2008.57 PMC2739676

